# Salicylic Acid Signalling in Plants

**DOI:** 10.3390/ijms21072655

**Published:** 2020-04-10

**Authors:** Tibor Janda, Gabriella Szalai, Magda Pál

**Affiliations:** Department of Plant Physiology, Agricultural Institute, Centre for Agricultural Research, H-2462 Martonvásár, Hungary; szalai.gabriella@agrar.mta.hu (G.S.); pal.magda@agrar.mta.hu (M.P.)

**Keywords:** acclimation, biosynthesis, cross talk, hormones, phenolics, plant growth and development, plant stress, salicylic acid, signalling

## Abstract

Ten articles published in the “Special Issue: Salicylic Acid Signalling in Plants” are summarized, in order to get a global picture about the mode of action of salicylic acid in plants, and about its interaction with other stress-signalling routes. Its ecological aspects and possible practical use are also discussed.

## 1. Introduction

Salicylic acid (SA) is ubiquitously distributed in the whole plant kingdom. The basal level of SA differs widely among species. It is generally present either in the free fraction or in the form of glycosylated, methylated, glucose-ester, or amino acid conjugates. In plants, SA can be synthesized via two distinct and compartmentalized enzymatic pathways, both requiring the primary metabolite chorismate. l-phenylalanine, derived from chorismate, can be converted into SA via the precursors free benzoic acid, benzoyl glucose, or *ortho*-hydroxy-cinnamic acid, depending on the plant species. Chorismate can also be converted into SA via isochorismate in the chloroplast. Several physiological processes in which SA may play a role have been reported, including seed germination, growth regulation, flower induction, thermogenesis, and especially, the regulation of plant responses under biotic or abiotic stress conditions. SA may be involved in different signalling processes. For example, various hormones involved in plant defence mechanisms crosstalk with SA, and both negative and positive interactions have been reported. SA signalling also leads to the reprogramming of gene expression and protein synthesis. It may affect the antioxidative metabolism, and it modulates cellular redox homeostasis. However, in spite of the extensive work on SA-related processes, the exact mode of action is poorly understood.

## 2. What Is Known So Far?

The effects and the role of SA in plant physiological processes have been widely studied for a long time. However, there are still a lot of open question in this field. This is also indicated by the fact that there are four review papers published on this topic, and they represent a wide range of approaches. These include a “classical” approach related to the role that SA plays in the presence of a stress factor—in this case, the heavy metal cadmium (Cd) [1]. Another review paper deals with another “classical” question: what is the mechanism of the SA perception, and which compounds are able to bind SA and transfer the message to further signalling routes [2]? The exact mechanisms related to SA are still poorly understood. This is also indicated by the fact that a relatively new approach, the involvement of SA in endoplasmic reticulum stress, has also been reviewed [3]. Finally, the last review addresses a possible practical use of SA, and its ecological consequences are also discussed [4].

To the best of our knowledge, heavy metal stress was one of the first processes where the protective role of SA in the case of an abiotic stressor was demonstrated. The treatment of cucumber or tobacco plants with SA induced tolerance against copper toxicity [5]. Similarly, SA treatment also ameliorated the effects Pb^2+^ or Hg^2+^ stresses in rice [6]. The pre-treatment of barley seedlings with SA reduced the oxidative damage caused by Cd, leading to increased biomass [7]. Cd is one of the most important pollutants, and may cause severe stress to plants, and through the food chain to humans, too. In order to reduce Cd injury, plants have evolved various cross-linked strategies, such as binding it to the cell wall [8], chelation with phytochelatins [9], and involvement of the antioxidant system [10].

The fact that SA signalling may also be a part of Cd tolerance is indicated by the fact that usually, exposure to Cd induces the endogenous SA level better than any other abiotic stressors [11]. Guo and co-workers recently reviewed the possible methods of application of exogenous SA to protect plants from Cd toxicity; they also reviewed the possible mechanisms that are induced by SA, and which play a role in the defence processes [1]. It is very important to emphasise that besides application mode, the effects of exogenous SA depend on various other factors, such as the concentrations of Cd and SA, the plant species, and the plant’s developmental stage. Based on various tested systems, Guo and co-workers concluded that the levels of SA in pre-soaking treatments or when used as foliar spray are generally higher than those in hydroponic treatments. This indicates that the uptake of SA through the root system is better regulated than via the leaves or the seeds [1]. The mode of action of SA during Cd stress is still poorly understood, and results are often controversial. In spite of the high number of successful applications of exogenous SA, results using mutant or transgenic *Arabidopsis* plants indicate that a high SA level may also be harmful [12]. However, using the *sid2* mutant supports the view that SA is necessary for efficient defence processes [13]. These results support the view that the SA level must also be fine-tuned in order to achieve the most effective protective effect. The defence against Cd is usually complex in plants, and it seems that SA affects various elements of it. SA has a dose-dependent effect on plant growth; however, while SA is also linked with other plant growth regulators, this effect may also be secondary, due to induction other protective mechanisms [14]. As a direct protective mode of action, SA may also increase the accumulation of Cd in the cell wall and prevent its translocation into other cell organelles. The possible effect of SA on the cell wall components has also been demonstrated on biotic stresses, where the cell wall is indeed the first line of the defence system [15]. As in the case of many others stressors, the induction of the antioxidant system seems a general mechanism to protect plants against the oxidative damage induced by excess levels of heavy metals. For example, in wheat, although a direct relationship was not found between the initial SA levels and the degree of Cd tolerance, the increased SA level in the root during cadmium stress was related to the enhancement of the internal glutathione cycle, thus inducing the antioxidant and metal detoxification systems [16]. However, these results cannot be generalized in many species, and most probably the novel role of SA in Cd toxicity will likely continue to be unveiled [1].

The study of SA-associated signalling pathways began almost simultaneously with the discovery of the role of SA in defence against pathogens. One of the most critical question was, and still is: what is (are) the main receptor(s) of the SA signal, and which proteins bind the stress-induced SA? NPR1 (nonexpresser of pathogenesis-related protein 1), as one of the salicylic acid binding proteins (SABPs), is a key transcriptional regulator of SA signalling. However, the many controversial data on the mode of action of SA indicates that SA signalling is not a simple “yes” or “no” question. Pokotylo and co-workers recently reviewed the possible groups of SABPs [2], listing a high number of different candidates. These include the well-known factors, such as catalase [17,18] or NPR proteins [19,20], as well as some others that are less frequently connected with SA signalling. Besides the characterization of the possible SABPs, it is also an important finding that although some SABPs are well-synchronised at the transcriptomic level, genes responsible for different SABPs can be both positively and negatively expressed in response to elicitation. Clarification of the interactions between the different regulating factors, and a better understanding the molecular mechanisms of interaction between SA and its binding proteins are among the main problems in the future.

The endoplasmic reticulum (ER) has multiple cellular functions, including protein synthesis. Unfavourable environmental conditions may disturb the ER homeostasis, causing ER stress [21]. This is accompanied by the accumulation of unfolded or misfolded proteins triggering the unfolded protein response. SA is a multifaceted compound, and it has been proposed that SA may also have role during ER stress. Poór and co-workers recently summarised the possible mode of action of SA in this process [3]. SA probably acts via regulating the redox homeostasis and inducing, directly or indirectly, specific transcription factors. Furthermore, the involvement of polyamines can also be assumed. Unfortunately, there are still a lot of open question in this field. Answering these questions about the role of SA and other phytohormones in ER stress and in the unfolded protein response may help to develop new strategies in agricultural research and practice, in order to protect plants against environmental stresses.

Present agriculture will probably have to face an important challenge soon: feeding the quickly increasing human population may require a second green revolution. Improvement of the stress tolerance of cultivated plants, and thus the increase of crop yields and nutritional values in environmentally friendly ways, is a crucial task in food production. Exogenous application of naturally occurring, biologically active compounds like SA can be an alternative approach to improving crop productivity under changing environmental conditions. The replacement of synthetic chemicals with natural plant secondary compounds could be an excellent option from economic and environmental points of view. With the application of these naturally occurring biologically active compounds, the acclimation processes can be intensified. However, there are still several open questions that must be answered before any of these compounds can be recommended in a responsible manner for practical use, especially under field conditions. The compounds’ effects must be known both for the plants where they are used, and for the wider environment. A recent review by Filgueiras and co-workers provides a detailed overview about the induction pathways induced by SA at the primary (genetic, metabolomic, and physiologic changes in plants), secondary (effects on herbivores and pathogens attacking the plants), and tertiary levels (with consequences for herbivore populations) [4]. For example, in addition to the investigation of the primary molecular effects of SA, another potential approach for the control of herbivores in agricultural systems through defences related to SA is attraction of natural enemies of the insects. The release of volatile SA-related compounds may also contribute to the reduction of the herbivore populations [22].

## 3. What Are the New Directions?

In spite of the intense research on the mode of action and possible practical use of SA, there are still a lot of open questions. Six research articles have also been published in the present “Special Issue”. These papers cover a wide range of aspects of SA-related mechanisms. They include a “classical” demonstration of the importance of SA in biotic stress [23] and the importance of the use of various mutants related to SA signalling [24,25], as well as focus on the crosstalk with other stress-related compounds [25,26]. There are also works investigating the effects of environmental factors on the SA-related signalling [26,27], and on the role of different SA analogue compounds [28].

The involvement of SA in biotic stress-related processes have been widely studied. However, Shi and co-workers were the first to demonstrate the responses of SA biosynthesis genes and molecular functions of SA in the response to anthracnose disease in tea plants [23]. This disease is induced by *Colletotrichum* fungi and may cause a 5–20% loss of tea yield. Data support the view that SA and its related signalling networks, including the induction of pathogenesis-related protein 1 (PR1) and connection with other plant hormones, play a pivotal role in the activation of tea immunity to anthracnose disease. They also provided a transcriptome dataset to profile gene expression and metabolic networks associated with tea plant immunity against anthracnose [23].

The fact that up to now, more than forty *Arabidopsis* mutants or transgenic lines with modified levels of SA or constitutively activated SA signalling pathways have been described also indicates the importance of SA signalling. The mutant collection presented by Pluhařová and co-workers provides a very valuable tool to better understand the mechanisms underlying trade-offs between growth and defence in plants. The authors present a novel research study, providing new insights clarifying a link between SA and plant behaviour under environmental stresses [24]. They showed a negative correlation between the SA content and the rosette size, but not the root growth. This is especially important, because hydroponically-added SA may often reduce root growth more than the shoots. Their results also draw attention to the importance of light intensity when data obtained under different growth conditions are compared.

SA signalling is not a simple linear route. SA may interact with several other stress-related compounds. The importance of polyamines in living cells has been known for several decades. However, their signalling role has only become apparent in recent years [29]. Tajti and co-workers recently demonstrated a possible crosstalk between the SA and polyamine signalling pathways [25]. They showed that the SA-deficient *Arabidopsis* mutant, *sid2* plants, could be characterised with a different polyamine metabolism. The *sid2* mutant plants also showed different responses to the exogenous polyamine treatments than the wild-type plants. Significant differences in the SA content and synthesis were also found between wild type and the SA-deficient, mutant *Arabidopsis* plants after polyamine treatments.

The efficiency of adaptation processes is influenced not only by genotype, but also by environmental factors. It has been shown that light during the acclimation period is required for the development of both the efficient freezing tolerance in frost-hardy cereals, and for the cold tolerance in the chilling-sensitive maize plants [30,31]. However, the interaction of light-mediated signalling and the development of cold tolerance processes is still poorly understood. A recent metabolomic research presented by Pál and co-workers demonstrated that different light conditions during the cold acclimation period differentially affected certain stress-related mechanisms in young maize plants, both in the root and in the leaves [26]. Chilling-induced accumulation of SA was light-dependent. The observed changes were also accompanied by hormonal and metabolic shifts.

SA signalling depends not only on the abiotic, but on the biotic environmental conditions too. It has been demonstrated that the inoculation of *Mentha x piperita* plants with different *Rhizobacteria* strains may increase the endogenous SA production [32]. Cappellari and co-workers have also now demonstrated that the inoculation of *M. piperita* with plant growth-promoting *Rhizobacteria* may modify the effects of exogenous application of SA or methyl jasmonate. Exogenous SA increased the total phenol content in this plant, and depending on the concentration used, certain *Rhizobacteria* could improve this effect. The exogenous SA also modified the production of the main monoterpene compounds [27]. These results suggest that a combination of SA or other plant growth regulator with certain plant growth-promoting *Rhizobacteria* may improve the production of secondary metabolites in aromatic plants.

It was suggested a long time ago that besides SA, some of its related compounds (synthetic ones or its natural precursors) may also have similar biological effects as SA itself [33]. Recently, Palmer and co-workers characterised several SA analogues, which were able to increase the strength of interactions among NPR3/4 in a yeast two-hybrid system. They induced NPR1 accumulation and the expression of PR1 in *Arabidopsis* plants, and were also able to inhibit the growth of plant pathogen bacteria, such as the citrus greening pathogen *Candidatus liberibacter* [28].

## 4. Conclusions

We hope that the reviews and studies published here in this special issue bring a closer understanding of the role of SA and an insight into its complex tasks, as well as a new direction for where there are still gaps and open questions that need to be explored, both at the metabolite and gene expression level, in the use of agriculturally important crop plants or mutant model plants, and not least of all, in both basic research and practical usage.

## Abbreviations

ER	Endoplasmic reticulum
NPR	Nonexpresser of pathogenesis-related
PR1	Pathogenesis-related protein 1
SA	Salicylic acid
SABP	Salicylic acid binding protein
